# Utilization of personal protective equipment and associated factors among building construction workers in Addis Ababa, Ethiopia, 2019

**DOI:** 10.1186/s12889-020-08889-x

**Published:** 2020-05-27

**Authors:** Addisu Alehegn Alemu, Meseret Yitayew, Aklilu Azazeh, Sofia Kebede

**Affiliations:** 1 College of Health Sciences, Debre Markos University, Debre Markos, Ethiopia; 2 College of Health Sciences, Assosa University, Assosa, Ethiopia; 3 Department of Emergency Medicine, School of Medicine, College of Health Sciences, Addis Ababa University, Addis Ababa, Ethiopia

**Keywords:** Personal protective equipment, Construction workers, Ethiopia

## Abstract

**Background:**

Personal protective equipment (PPE) is a material, device, equipment, or clothing used or worn by workers to reduce their chance of exposure or contact with any harmful material or energy that causes injury, disease, or even death. The use of PPE is a universal legal requirement to reduce occupational injuries and illnesses in the workplace. Therefore, this study was conducted to assess PPE utilization and its associated factors among building construction workers in Addis Ababa, Ethiopia, 2019.

**Methods:**

Institution based quantitative cross-sectional study was conducted on the selected construction sites of Addis Ababa city from April 1 to May 18, 2019. Data were collected among all (206) building construction workers via interviewer-administered questionnaires. Data were entered into Epi info version 7.1 and exported to SPSS version 25 statistical software for analysis. Variables with a *p*-value of less than 0.20 in bivariate analysis were included in multivariate logistic regression. Finally, variables with a p-value of less than 0.05 in multivariate analysis were considered as significantly associated.

**Results:**

The utilization of at least one PPE among building construction workers in Addis Ababa was found to be 38%. The majority (41.1%) of the participants’ reason for not using PPE were the unavailability of PPE followed by absence of orientation on using PPE (21.3%). The majority (35.3%) and (32.2%) of participants knew abrasion as a type of injury and suffered from abrasion respectively. Factors associated with utilization of PPE were the presence of training on PPE use (AOR = 4.8; 95% CI: 2.3, 10.3), presence of safety training (AOR = 2,8; 95% CI:1.5, 5.2), safety orientation before commencing work (AOR = 4.0; 95% CI:1.9, 9.0) and presence of supervision (AOR = 5.0; 95% CI:1.9,13).

**Conclusions:**

PPE utilization among building construction workers in Addis Ababa was low. The main reasons for non-utilization of PPE were unavailability of the materials and the absence of orientation on using PPE. The presence of PPE use training, presence of safety training, safety orientation, and governmental supervision were factors associated with PPE utilization. There should be continuous supervision of construction sites to assure all workers get material and training on how to use it.

## Background

Personal protective equipment (PPE) is any equipment used to eliminate or minimize a specific occupational injury by a worker [1–3]. Its utilization is a universal, legal requirement and recommended action of workers to protect themselves from injuries in their workplace [3, 4]. Though the occupational injury is a global public health problem, it is the worst in the developing countries but can be prevented through effective use of the necessary PPE during work [3, 5, 6].

According to the International Labor Organization (ILO) and World Health Organization (WHO) 270 million occupational accidents [6],160 million new cases of occupational diseases respectively are caused annually by exposure and dangerous conditions at the workplace; 30–40% of these can end up with chronic diseases, permanent disability, pulmonary impairment and premature death [5, 7–11]. Building construction is a complex sector where injuries at the workplace are enormous [3] and are hurtful to the employee, the employer, and society [5]. The probability of a fatal accident in the construction industry is five times the probability of an accident in the manufacturing industry [12].

The impact of occupational health and safety hazards faced by construction workers in developing countries is 10 to 20 times higher than those in industrialized countries [13]. Workers in the construction sectors are more exposed to physical, psychological, biological, and chemical risk factors [14]. Safety issues in the construction industry receive inadequate attention and provision in the developing world. This is due to owing to cost, lack of enforcement, and ignorance of the stakeholders [15]. Occupational injuries pose major public health and developmental problems; which result in serious health, social, and economic consequences on workers and their employers [5]. Employees must understand when is necessary to use, what equipment is required, how to use or wear, how to care, how to know when the equipment has reached the end of its useful life and how to dispose of PPE [6, 16, 17]. The prevalence of occupational injury is high which is by low PPE usage [18].

There are huge and numerous building construction projects with many occupational injuries (including death) in Addis Ababa, Ethiopia, however PPE utilization and its determinants among building construction workers are not well known. Therefore, this study identified the use of PPE and its determinants among building construction workers in Addis Ababa, Ethiopia.

## Methods

### Study area, design, period and population

A facility-based quantitative cross-sectional study was conducted at selected construction sites of Addis Ababa City that have direct regulatory communication with Addis Ababa city administration construction office. The study was conducted to assess PPE utilization and its determinants among building construction workers from April 1 to May 18, 2019. Addis Ababa is the capital and largest city of Ethiopia and a head seat for the African Union, home for more than 92 embassies and consular representatives and international agencies. Among numerous construction companies in Addis Ababa, only five of them were regulated by the government [5]. This study included all construction sites that had direct regulation with Addis Ababa construction office during the study period. All construction site workers in these sites were included.

### Sample size determination and sampling procedure

**Fig. 1:**
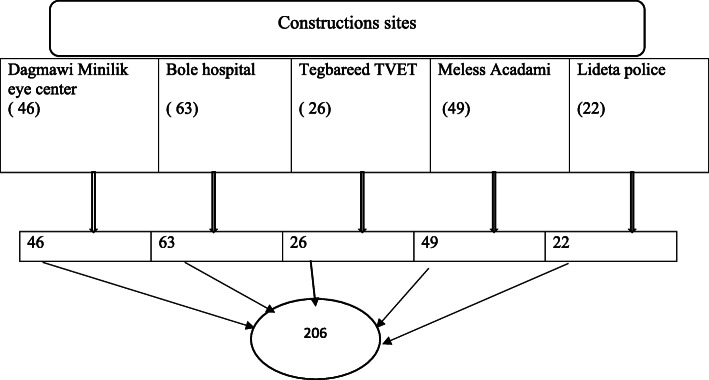
A selection of study participants for PPE utilization among building construction site workers in Addis Ababa, Ethiopia, 2019

All construction site workers (206) in the five sites who were functional and regulated directly by Addis Ababa construction office during the study period were included in this study (Fig. 1).

### Data collection technique and study variables

Data were collected using translated structured Amharic questionnaires developed after intensive review of previous studies related to this study. Data were collected using face-to-face interviews. Questionnaires included socio-demographic, behavioral, and environmental factors that can affect PPE utilization. Data were collected by six BSc health professionals under the supervision of two MSc candidates.

### Data quality, processing, and analysis

Proper designing and pretesting of the questionnaire in 5% (11 construction workers) of the participants was conducted in one building construction site other than the selected sub-city (Akaki). Two days of training before and after the pretest was given to data collectors and supervisors. They were trained about the objectives of the study, the questionnaire’s content, the data collection techniques, and the way how confidentiality maintained throughout the study. In addition, there was were daily checking of the incompleteness and inconsistencies of questionnaires. The collected data were entered into Epi Info version 7.1 (Atlanta, GA) and exported to SPSS version 25 (Armonk, NY: IBM Corp) for statistical analysis. During analysis, those variables in bivariate analysis with a *p*-value of less than 0.20 were included in multivariate logistic regression. Finally, multivariate analysis was computed and variables with p-value less than 0.05 were considered as significantly associated.

### Definition of terms

#### Personal protective equipment

Any material, device, equipment or clothing which is used or worn by a worker to protect them from exposure or contact with any harmful material or energy which may cause injury, disease or even death to the worker, such as earplugs, Googles, Overalls, gloves, helmets and safety shoes [19].

#### PPE utilization

The use of at least one PPE consistently and appropriately to prevent injuries and illness.

## Results

### Socio-demographic characteristics

**Fig. 2:**
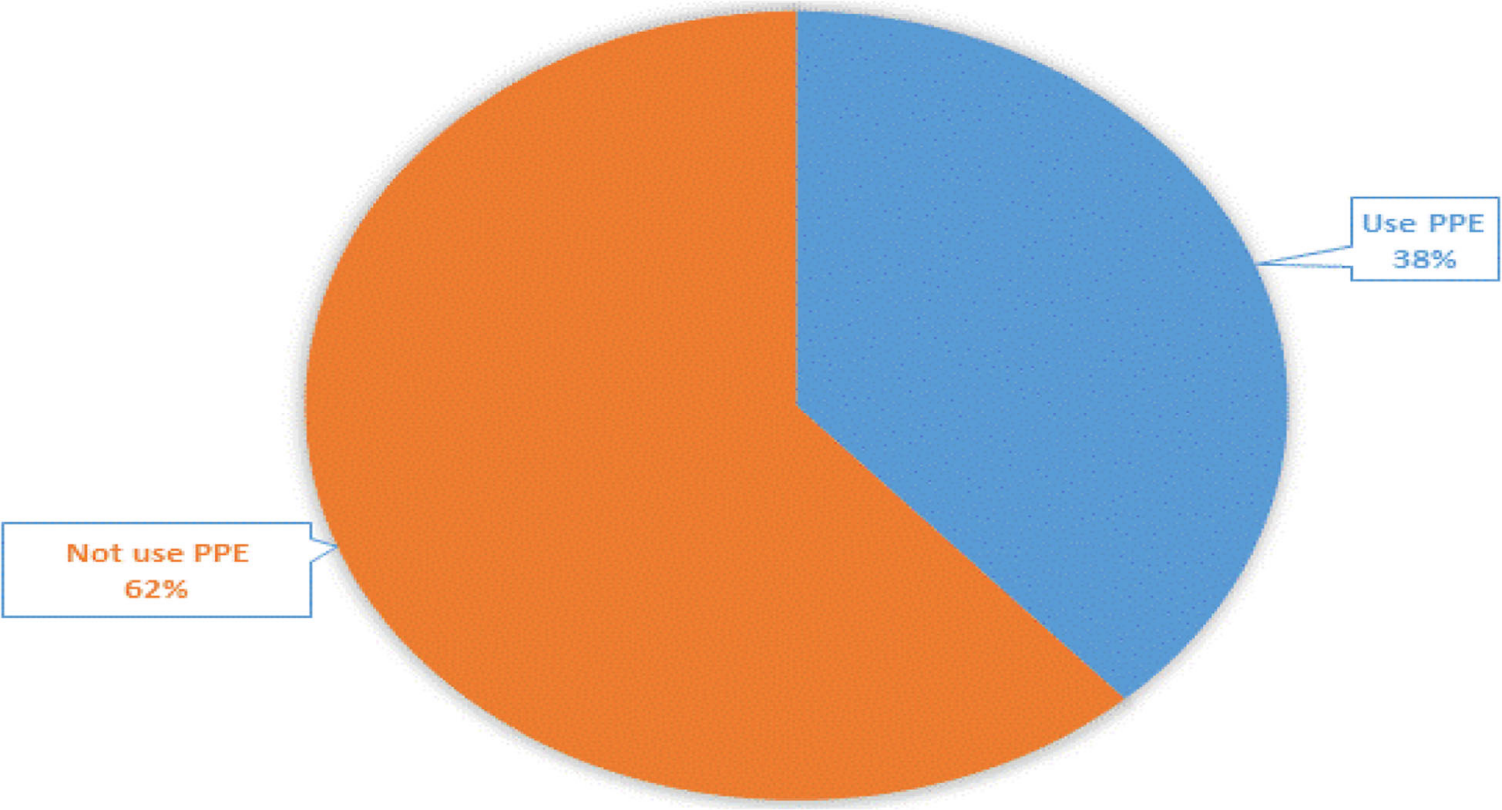
PPE utilization among building construction site workers in Addis Ababa, Ethiopia, 2019

A total of 206 respondents were interviewed with a response rate of 100%. This study showed that 62% of participants had not used even a single personal protective equipment (Fig. 2). Three in 10 of the respondents were female. The mean age of respondents was 25.5 (SD + 4.07) years. The majority (88.3%) of respondents were below 30 years old. The study also showed six in 10 were single. Three in 10 had no formal education, whereas, 26.2 and 23.8% of the participants had primary and secondary educational status, respectively. The majority (71.4%) of workers were recruited on a daily basis (Table 1).

**Table 1:** Socio-demographic characteristics of building construction workers in Addis Ababa, Ethiopia, 2019

Variable	Category	Frequency	Percent
Sex	Male	157	76.2
	Female	49	23.8
Age	<=30	182	88.3
	> 30	24	11.7
Marital status	Married	81	39.3
	Single	122	59.2
	Divorced	3	1.5
Working hour	< than 5 h	7	3.4
	5–6 h	7	3.4
	7–8 h	162	78.6
	9–10 h	19	9.2
	11–12 h	11	5.3
Working experience	≤ 1 year	123	59.7
	> 1 & < 5 years	63	30.6
	≥5 & < 10 years	15	7.3
	≥10 years	5	2.4
Term of employment	Fixed contract	59	28.6
	Daily	147	71.4
Level of education	No formal education	62	30.1
	Primary	54	26.2
	Secondary	49	23.8
	College / Above	41	19.9

### Reasons for not utilizing PPE

This research identified the reasons for not using PPEs. The majority of participants (41.1%) reported they did not use PPE due to the unavailability of PPE, followed by (21.3%) due to lack of orientation on PPE usage. Similarly, 16.3, 11.3, and 10% of participants reasons for not using were the PPEs were not comfortable, PPEs are not important and have no reason at all, respectively.

### Type of injuries known and suffered by the participants

This study identified that the most commonly perceived type of injury was abrasion (35.3%) and only 2% of the participants perceived permanent disability at the building construction site. Almost one-third of participants suffered abrasion whereas one or two out of 10 participants suffered from a bone fracture. Multiple causes of injury and more than one perception were identified (Table 2).

**Table 2:** Type of injuries known and suffered among building construction site workers in Addis Ababa, Ethiopia, 2019

Variables		Frequency	Percent
Type of injury known	Abrasion	140	35.3
	Piercing/Cut	93	22.7
	Muscular pain	71	18
	Bone fracture	46	11.5
	Sprain/strain	39	10.5
	Permanent disability	10	2
Type of injury suffered	Abrasion	109	32.2
	Piercing/Cut	104	30.8
	Bone fracture	46	13.6
	Sprain/Strain	34	10.1
	Muscular pain	26	7.7
	Back pain	19	5.6

### Preparedness for injury prevention

In this study preparedness of injury prevention was assessed, the majority (84.5%) of participants knew at least one type of PPE, only nearly one third (31.6%) the participants took safety training and around four out of 10 participants had been oriented about PPE before commencing their work (Table 3).

**Table 3:** Preparedness for injury protection among building construction workers in Addis Ababa, Ethiopia, 2019

Variable		Frequency	Percent
Knows any PPE	Yes	174	84.5
	No	32	15.5
Took safety training	Yes	65	31.6
	No	141	68.4
Orientated about PPE	Yes	73	35.4
	No	133	64.6

### Factors associated with PPE utilization

Independent variables like age, level of education, the term of employment, year of experience, availability of safety training, PPE utilization training, perception of injury, knowledge on PPE, safety brief before commencing work and availability of enforcement or pressure to use PPE was tested by binary logistic regression. Variables with a *p*-value of less than 0.2 during the bivariate analysis were included in the multivariate logistic regression. Based on this, safety training, PPE use training, safety brief before commencing work, and governmental visit showed association with PPE utilization. Those who took general safety training on PPE use were about 3 times more likely to use personal protective equipment (AOR = 2.8; 95% CI: 1.5, 5.2) than those who had not. Those who had been briefed about safety before commencing work were 4 times more likely to use personal protective equipment (AOR = 4.0; 95% CI:1.9, 9.0). Those who had PPE use training are 4.8 times more likely to use PPE (AOR = 4.8; 95% CI: 2.3, 10.3) and workers in the sites where there was supervision by the governmental concerned body were 5 times more likely to use PPE AOR = 5.0; 95% CI:1.9, 13) than who did not (Table 4).

**Table 4:** Factors associated with personal protective equipment utilization among building construction workers in Addis Ababa, Ethiopia, 2019

		PPE use			
Variable		Yes	No	COR (95%CI)	AOR (95%CI)
Age	≤ 30	73	109	2.01 (0.76,5.30)	0.90 (0.24,3.39)
	> 30	6	18	1	1
Level of education	Illiterate	18	44	1.73 (0.75,3.96)	2.37 (0.8,7.02)
	Primary	20	34	1.20 (0.52,2.76)	1.65 (0.56,4.87)
	Secondary	24	25	0.74 (0.32,1.70)	1.01 (0.34,2.97)
	> College	17	24	1	1
Term of employment	Fixed contract	18	41	0.62 (0.33,1.18)	0.72 (0.30,1.68)
	Daily	61	86	1	1
Year of experience	< 1	45	78	1	1
	1–5	24	39	0.40 (0.04,3.85)	0.55 (0.42,7.02)
	6–10	9	6	0.17 (0.01,1.88)	0.25 (0.02,3.93)
	> 10	1	4	0.43 (0.05,4.00)	0.47 (0.38,5.64)
Safety training	Yes	36	29	2.83 (1.54,5.19)***	2.30 (1.08,5.06)^*^
	No	43	98	1	1
PPE use training	yes	48	27	5.73 (3.08,10.66)***	4.80 (2.26,10.27)^***^
	No	31	100	1	1
Perceives Any Injury	Yes	75	111	1	1
	No	4	16	0.37 (0.12,1.15)	0.87 (0.20,3.64)
Suffer Injury	Yes	70	100	1	1
	No	9	27	0.47 (0.21,1.07)	0.77 (0.26,2.26)
Knows any PPE	Yes	74	100	1	1
	No	5	27	0.25 (0.92,0.68)**	0.48 (0.13,1.67)
Orientated about PPE	Yes	44	29	4.25 (2.31,7.79)***	4.00 (1.92,8.98)^***^
	No	35	98	1	1
Presence of supervision	Yes	27	9	6.80 (2.99,15.49)^***^	5.00 (1.93,13.10)^***^
	No	52	118	1	1

*Significant codes*: *** < 0.001 ** < 0.01 * < 0.05

## Discussion

Occupational injuries which are the global public challenges [20] are common among building construction workers [21]. All workers in the construction industry should be trained on the proper use of PPE [22] to get them protected from potential occupational hazards [23]. Determining factors associated with PPE utilization among workers is vital for implementing the safety work promotion programs [3]. The purpose of this study was to determine the utilization of PPE and its associated factors among building construction workers in Addis Ababa, Ethiopia. The study included only construction sites directly regulated by the Addis Ababa construction office were included to identify the PPE utilizations though they were regulated.

In this study, the PPE utilization among construction site workers was 38%. The utilization of PPE in this study is in line with a study conducted in Cairo, Egypt (31.4% [24], but lower than studies conducted in Hawassa, Ethiopia (82.4%) [25] and Kampala, Uganda (50.4%) [26]. This might be due to differences in methodological differences, study populations, and study areas. The two main reasons for not using PPE identified in this study were unavailability of PPE (41.1%) and lack of orientation on how to use PPE (21.3%). Similar studies also identified the reasons for not using PPE was the unavailability of PPE [23] uncomfortable, not designed for hot and cold weather, and heaviness [22, 27, 28]. Other reasons might be due to lack of interest and awareness from workers, lack of concern from responsible bodies, and uncomfortable PPE.

This study revealed safety training as a significant factor of PPE utilization. Workers who have not taken safety training were less likely to utilize PPE. This was supported by previous studies [8, 29–36]. Similarly, those who had not been trained on PPE utilization were less likely to utilize PPE in line with the previous studies [32, 36]. On the other hand, inadequate or lack of training on PPE usage shows low PPE utilization [37]. This might be due to the reason training workers on safety and proper PPE use promotes [34, 38]. It has paramount advantages on appropriate utilization of PPE [39] which is intended to prevent the risk of worksite injuries [40, 41].

This study, similar to previous studies [30, 31, 42–44], identified supervision on PPE usage was significantly associated with PPE utilization. Those who had been supervised on their PPE usage were 5 times more likely to utilize PPE compared to those who had not been supervised. The reason behind this could be that workers who are supervised and reminded of the applications of PPE [24] might be enforced to use PPE with fear of not being subjected for criticism or even days or total off [43]. Additionally, this study showed safety orientation before commencing work was a significant factor affected PPE utilization. Those who had been oriented before commencing work were 4 times more to use PPE than those who had not been oriented. This finding is supported by previous studies [45, 46].

### Limitation of the study

Since the list was taken from the employers, workers who might be absent from work due to injury might not be included in this study.

## Conclusions

This study showed only 38% of workers utilized PPE. The majority of respondents’ reason for not using PPE was the unavailability of PPE. Abrasion and piercing /cut were a common type of injury and clamping at high and dust/fug are common reasons. There should be training on PPE use before commencing work to strengthen workers’ safety and improve the productivity of manufacturing industries.

## Data Availability

The data that support the findings of this study are available from the corresponding authors upon reasonable request.

## Abbreviations

AAU: Addis Ababa Universit
ILO: International Labour Organization
OSHA: US Occupational Safety and Health Administration
PPE: Personal Protective Equipment
UN: United Nation
WHO: World Health Organization
COR: Crude odds ratio
AOR: Adjusted Odds Ratio
CI: Confidence interval
